# Climate Change, Northern Birds of Conservation Concern and Matching the Hotspots of Habitat Suitability with the Reserve Network

**DOI:** 10.1371/journal.pone.0063376

**Published:** 2013-05-20

**Authors:** Raimo Virkkala, Risto K. Heikkinen, Stefan Fronzek, Niko Leikola

**Affiliations:** 1 Finnish Environment Institute, Natural Environment Centre, Ecosystem Change Unit, Helsinki, Finland; 2 Finnish Environment Institute, Climate Change Programme, Helsinki, Finland; 3 Finnish Environment Institute, Natural Environment Centre, Biodiversity Unit, Helsinki, Finland; Università degli Studi di Napoli Federico II via Università, Italy

## Abstract

National reserve networks are one of the most important means of species conservation, but their efficiency may be diminished due to the projected climatic changes. Using bioclimatic envelope models and spatial data on habitats and conservation areas, we studied how efficient the reserve network will be in preserving 100 forest, mire, marshland, and alpine bird species of conservation concern in Finland in 2051–2080 under three different climate scenarios. The occurrences of the studied bird species were related to the amount of habitat preferred by each species in the different boreal zones. We employed a novel integrated habitat suitability index that takes into account both the species’ probability of occurrence from the bioclimatic models and the availability of suitable habitat. Using this suitability index, the distribution of the topmost 5% suitability squares (“hotspots”) in the four bird species groups in the period 1971–2000 and under the three scenarios were compared with the location of reserves with the highest amounts of the four habitats to study the efficiency of the network. In species of mires, marshlands, and Arctic mountains, a high proportion of protected habitat was included in the 5% hotspots in the scenarios in 2051–2080, showing that protected areas cover a high proportion of occurrences of bird species. In contrast, in forests in the southern and middle boreal zones, only a small proportion of the protected habitat was included in the 5% hotspots, indicating that the efficiency of the protected area network will be insufficient for forest birds in the future. In the northern boreal zone, the efficiency of the reserve network in forests was highly dependent on the strength of climate change varying between the scenarios. Overall, there is no single solution to preserving biodiversity in a changing climate, but several future pathways should be considered.

## Introduction

Climate change is a major threat to biodiversity [1], [2], which puts pressures on species to move to new climatically suitable areas [3]–[5] and highlights the need for designing conservation strategies for climate-change adaptation [6], [7]. In such strategies, the network of protected areas is one of the most important means of enhancing species survival [8], [9]. However, novel challenges to the efficiency of the reserve network are evident, because the changing climate may drive species outside the protected areas which they currently occupy [10]–[15].

The efficiency of the protected area network in preserving biodiversity is the main point for the conservation biology communities, i.e. identifying sites with the highest conservation value, the establishment and management of protected areas and the implementation of suitable conservation measures [16]–[18]. Numerous studies have used modelled projections of the range shifts of species under climate change scenarios, accompanied by conservation planning tools that address species’ present and potential future distributions [10], [12], [14], [19], [20]. Such studies have provided important insights into potential species losses, turnover and gain in conservation areas, as well as future gaps in the reserve system. In general, the studies employ bioclimatic envelope models (BEMs), whereby the relationships between present-day distributions of species and climatic variables are modelled and then used to forecast the future changes in a suitable climate space for species [21]–[25].

However, many climate change studies assessing reserve network impacts, as well as basic BEM studies, have methodological limitations which may weaken the accuracy of assessments they provide. First, most studies have converted the probability of occurrence data for present-day and future conditions into plain presence/absence data based on a certain cut-off level, such as prevalence [26]–[28], maximized Cohen’s Kappa [5], [12], [23], [29] or threshold optimizing the percentage of correctly predicted absences and presences [11], [30]. However, the use of one single cut-off level deeming sites as either climatically suitable or unsuitable may exaggerate the effect of climate shifts on species’ future distributions [31], and result in the lumping of drastic changes in climatic suitability with marginal changes [32].

Second, some studies have addressed the difficulties in determining how much conservation area there should be in a grid cell of a given size so that a modelled species can be considered as protected [14], [29], [33], [34]. However, earlier BEM studies dealing with climate change impacts have predominantly employed only the total size of the conservation areas [14], [29], [30], [35]; it has been surprisingly rarely investigated how much preferred habitat actually exists for the focal species in the conservation areas modeled as (remaining or becoming) suitable in the face of climate change. This runs the risk of making false assumptions about the availability of protected habitat for a given species in a given locality, and obstructs the ranking of areas for conservation planning under changing climate based on the amount of preferred habitat [36]. Alongside with this limitation, there is a long history of assessments and modelling of the species’ biogeographical hotspots, i.e. areas of high concentration of species [37]–[39]. Some BEM studies have addressed how severely species ranges will be reduced in the regional hotspots [31], [40]–[42], or assessed future biases of the reserve network in global biodiversity hotspots (e.g. [43]). However, very little attention has been paid to the amount of suitable protected habitat. In fact, extremely few studies have considered the joint importance of the amount of preferred habitat and the degree of climatic suitability in the conservation areas across a range of species to determine potential species hotspots and the spatial alterations of such hotspots under a changing climate (for a solitary exception see [44]).

Third, studies have mainly focused on the projected future changes in species occupancy patterns and hotspots in the reserves themselves and not in the adjacent non-protected areas (but see [10], [11], [15], [35], [41]). By and large, what appears to be missing is analysis that compares the hotspots of joint habitat availability – climatic suitability for multiple species in reserves with corresponding hotspots in the more poorly protected areas, to assess reserve network efficiency and determine sites for potential new conservation areas.

Progress in this arena has been made recently but generally just in one of these three topics. Some studies have retained probability values higher than the threshold to assess the relative differences in climatic suitability of the grid cells for the modelled species instead of using plain converted species present/absent information (e.g. [13], [14], [45]). In the other topics, some work has been targeted to either filter out the human-transformed degraded areas from future projections of species [15], [41], link the climatically suitable areas with suitable land cover types [30], and to delimit potential future agglomerations of species occurrences in non-protected landscape [10], [11], [19]. In an interesting study, Marini et al. [35] used the BEM-ensemble modelling approach to identify areas with high future cumulative probability of occurrences for bird species and potential major gaps in the reserve system.

However, in order to add an important dimension of accuracy into species – climate change based conservation planning, we need to address all three shortcomings simultaneously. Here we make such an attempt by developing BEMs for 100 Northern European bird species of conservation concern and projections of climatic suitability for Finland at present and in the future, and by taking into account the amount of preferred habitat for the modelled species in protected and unprotected areas. Our set of bird species is a useful model system, because their distributions are well covered both in national Atlas and European-wide Atlas compilations (see [46], [47]). Several BEM studies (e.g. [5], [20], [24], [45], [48], [49]) have been conducted based on them. Moreover, it is anticipated that northern latitudes will face greater projected increases in temperature than most other parts of the globe [50]–[52] which may give rise to notable changes in species ranges [24], [49] and challenge the efficiency of the reserve network.

In a previous study [36] we employed the same bird species and climate data, and investigated whether there are differences in the projected changes in the climatic suitability for the species in grid cells with highest amounts of protected preferred habitat vs. grid cells with similar amounts of unprotected habitat. In the present study, we use the same study setting to introduce an important new dimension into our assessment. Here we will specifically develop a joint index of the amount of preferred habitat and projected probability of occurrence for each species, in order to locate the areas with maximal suitability for the species, both presently and in the future climate, and compare these species-level hotspots of suitability with the locations of reserves with a high amount of suitable habitat. Moreover, we will generate an overall suitability-hotspot index to determine areas where high suitability coincides for many species. These analyses will be performed separately for four main terrestrial species groups, i.e. species from forests, mires, marshlands and Arctic mountain habitats. The trends emerging from these groups will be compared to address whether the conservation status of the hotspots of suitability will show different projected trends, indicating higher future conservation needs in some species groups.

## Materials and Methods

Most of the material and methods underlying this study with the exception of the habitat suitability analyses, have been described in more detail in Virkkala et al. [36] and thus are only briefly presented here.

### Bird Species

We included 100 land bird species of conservation concern in the study, of which 51 were regarded as species of forests, 21 species of mires, 17 species of marshlands and 11 species of Arctic mountain habitats (Table S1). We took into account both regularly breeding species (90 bird species) in Finland, and also species of conservation concern (eight forest species and two marshland species) that currently do not occur in Finland but are breeding in adjacent regions to the south or south-east of Finland.

The 100 studied species were selected using a number of classifications of conservation concern and the critical categories in them (Table S1): the European Union's Birds Directive species (Annex I), species of European conservation concern (SPEC1–SPEC3) [53], species of Arctic or boreal biome for important bird areas in Europe (IBA) [54], threatened species in the European Union (unfavourable conservation status) [55], species of special responsibility in Finland [56], red-listed species in Finland in 2010 (near-threatened and threatened species) [57], and species preferring old-growth or mature forests in Finland [58]–[60]. Species in the study belonged to at least one of these classifications. Species of agricultural habitats or human settlements, and species of lakes (e.g. waterfowl) or the Baltic Sea were not included in the analysis because the study was carried out in the terrestrial protected area network, which consisted of forests, mires and other wetlands (here regarded as marshlands) and mountain habitats.

### Habitat Classification and Protection of Habitats

Habitats and protected areas were investigated separately in three main vegetation zones in Finland: the southern boreal, the middle boreal and the northern boreal zones. The extent of the hemiboreal zone in the south-western coast of Finland was small, and therefore it was included in the southern boreal zone. In the north, mountain birch forms both the northernmost forests and the tree line in the northern boreal zone.

The proportion of land cover types was calculated as a cover for each 10×10 km cell and also specifically within each of the conservation area included in the study from the digital CORINE Land Cover 2006 database (resolution 25 meters), using ArcView Spatial Analyst (Version 3.2, ESRI, Redland, CA, USA). Different coniferous, deciduous and mixed forest classes were combined as a single forest class except mountain birch woods above the coniferous forest boundary in the northern boreal zone. Open mires (i.e. treeless peatlands) were regarded as mire class. Following Virkkala et al. [61], wetlands along sea and lake shores and along rivers and in estuaries were regarded as marshland habitats (largely dominated by the common reed *Phragmites australis*). Open, treeless Arctic mountain habitats in the northern boreal zone were regarded as mountain heaths. Each of the 100 bird species were assigned to one of the four habitat categories based on their main habitat in Finland or areas south of Finland (Table S1).

**Figure 1:**
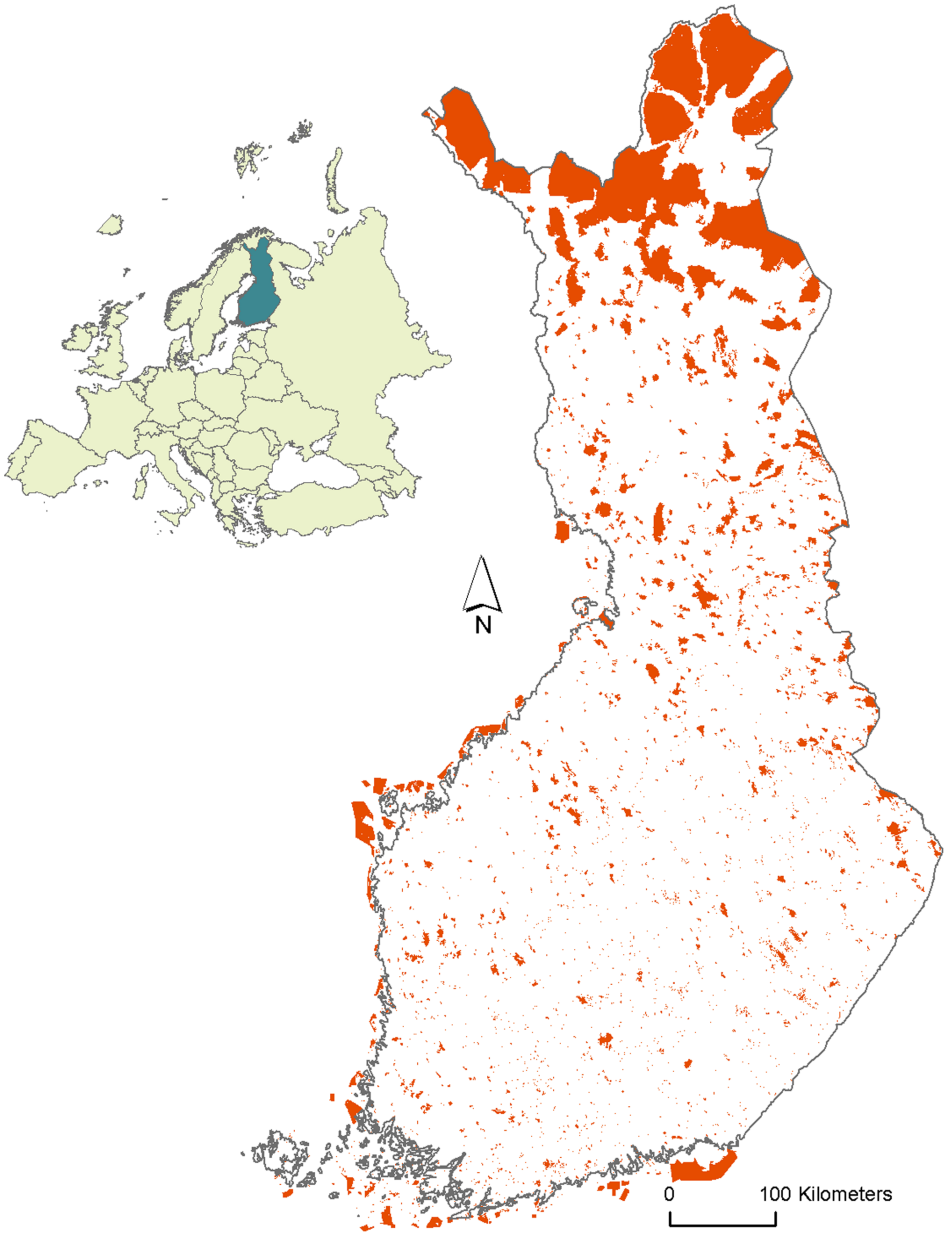
Protected area network in Finland.

The amount of protected habitat differs considerably both from south to north (see Fig. 1) and between-habitat types [36]. Some 2.3%, 3.7% and 23.4% of all forests were situated in protected areas in southern boreal, middle boreal and northern boreal zones, respectively. For mires the corresponding figures were 19.0%, 19.4% and 35.2%, and for marshlands 26.7%, 24.2% and 12.1%, respectively. In Arctic mountain heaths, 88.9%, and in mountain birch woods, 82.4%, were included in protected areas.

### Species Atlas Data and Climate Data

We used the European Bird Atlas, the EBCC Atlas for European Breeding Birds [46], in developing the bioclimatic envelope models for the 100 bird species studied. We focused on present-absent data from all the European Bird Atlas 50-km grid squares where data covered at least 75% of expected breeding species (see [46]). A species was considered to be breeding in a grid cell when the record for it fell under one of these categories: possible, probable and confirmed breeding.

Following earlier studies [27], [48], [49], [62], we selected five climate variables as potential explanatory variables for BEMs: mean temperature in April–June (TEMP_AMJ_ ), mean temperature of the coldest month (MTCO), annual daily temperature sum above 5°C (growing degree days, GDD5), mean precipitation in April–June (PREC_AMJ_), and mean annual precipitation (PREC).

An observed Eurasian-wide climate dataset of monthly mean temperatures and precipitation at 10′×10′ spatial resolution averaged for the period 1971–2000 was constructed by combining two gridded databases: the CRU_TS_3.1 dataset at 30′×30′ grid resolution with monthly data during the period 1961–2000 (updated from Mitchell and Jones [63]), and monthly climatologies for the period 1961–1990 at 10′×10′ grid resolution from the CRU_CL_2.0 dataset [64]. Data were extracted for Eurasia for the window 12°W to 70°E longitude and 34°N to 72°N latitude consisting of 73,670 grid cells at 10′ resolution and 9,167 grid cells at 30′ resolution (for more details, see [36]).

Climate data in 10′ resolution were related to the 50×50 km grid system used in the EBCC Atlas for European Breeding Birds by calculating the mean, minimum and maximum for TMP_AMJ,_ MTCO and GDD5 of all 10′ grid cells whose centre point was located within a given 50×50 km cell. The minimum and maximum values were applied so that our bioclimatic envelope models would include the preferences of bird species favouring lowland or upland climate conditions. Overall, 11 individual climate variables were considered in the model building.

Three climate scenarios representing a wide range of possible climatic conditions for 2051–2080 were constructed for our climate variables on a 10×10 km grid covering Finland. Climate data for an ensemble of 19 General Circulation Models employed in the IPCC’s Fourth Assessment Report [65] (GCMs; for the selected models, see Virkkala et al. [36]) were downloaded from the archive of the Third Coupled Model Intercomparison Project (CMIP3; [66]). In addition to the central ensemble scenario, two individual GCM simulations representing low and high changes were selected. Thus, we included: (i) the 19-model ensemble mean for the SRES A1B emission scenario representing average changes; (ii) a simulation of the Australian CSIRO-MK3.0 GCM for the SRES B1 (from now on B1) scenario, which shows relatively low annual temperature and precipitation changes across Finland throughout the 21st century; and (iii) a simulation of the Japanese MIROC3.2/medres GCM for the SRES A2 (from now on A2) scenario, which is among those simulations giving higher annual temperature and precipitation increases. We calculated simulated changes in temperature and precipitation from these scenarios between the baseline and scenario periods and applied these to an observed monthly climatology on a 10 km×10 km grid for Finland.

### Statistical Analyses

The European-wide climate and bird data were employed to calibrate the bioclimatic models. The mean, maximum and minimum values for TMP_AMJ_, MTCO and GGD5 were highly intercorrelated. Thus, we first selected either the mean, maximum or minimum value for each study species and each of the three variables. This was done using generalized additive models (GAM) via the GRASP user-interface and the measures of predictor variables importance therein, see [36], [67]. Next, a second GRASP run was conducted where we included the remaining five climate variables, and in if high (>0.9) correlations between the five predictor variables appeared, a variable with lower importance was excluded. However, for the precipitation variables we used only the mean values of the 10′×10′ grid cells. Moreover, because the correlation between precipitation variables and the other variables was always below <0.8 (cf. [68]) we included these two variables in all our BEMs.

The calibrated bioclimatic envelope models with the European climate (1971–2000) and bird data (50×50 km) were first projected for the climate data for Finland averaged over the period 1971–2000 in the 10×10 km grid scale. This provided the baseline estimate of the climatic suitability (i.e., predicted probability of occurrence) of each 10×10 km grid cell for each bird species. Next, we fitted the derived models to the three climate scenario datasets for the time period 2051–2080, which provided estimates of the future climatic suitability for each species.

The bioclimatic envelope models were generated using the BIOMOD framework (the version described in [69] in the R environment version 2.12., and the three modelling techniques, generalized linear models (GLM), generalized additive models (GAM) and generalized boosting method (GBM). For each of the 100 bird species, polynomial GLMs were computed using an automatic forward stepwise procedure together with the Akaike information criterion (AIC) model selection criteria. To produce GAMs, we used a cubic spline smoother with three degrees of freedom, and the step forward variable selection of GAM in BIOMOD based on AIC was employed [70]. GBM was implemented into R (R Development Core Team, 2011) [71] using the library GBM (Generalized Boosted Regression Modelling). The maximum number of trees was fixed at 2,000, and a four-fold cross-validation procedure was employed. For more details on the modelling methods used here, see [67], [72]–[78].

We assessed the predictive power of the derived models using a four-fold cross validation with a random split into 20% validation and 80% calibration datasets. The discrimination ability of the predictive models was determined using three commonly used measures, which were: 1) the area under the curve (AUC) of a receiver operating characteristic (ROC) plot [79], 2) the kappa statistic [80], and 3) the true skill statistic (TSS; Allouche et al. [81]). The predictive performance of all our BEMs (three modelling techniques times 100 species) was generally good or excellent, as only two species showed AUC values lower than 0.80, see [36].

Based on the probability of occurrence of species generated by the three modelling methods, the arithmetic mean probability value was calculated for each species in each 10-km square for the periods 1971–2000 and 2051–2080. In calculating the joint habitat suitability index (joined species-specific and joined overall habitat suitability index), the amount of focal habitat (forest, mire, marshland and Arctic mountain habitats) in each square was first standardised to vary between 0 and 1 by dividing it by the largest amount of habitat per square in the corresponding zone. This was done because the amount of different habitats varies considerably in Finland (see [36]): the total land area of forests is 184,854 km^2^ (60.7% of total land area of 304,474 km^2^ in Finland) and that of marshlands 963 km^2^ (0.3%). Then this rescaled habitat amount was multiplied by the species probability of occurrence (values 0–1) to produce the joined species-specific habitat suitability index in each square for each species separately. Thus, in this procedure each species was related to its species-specific habitat, i.e. forest species to forest habitat, mire species to mire habitat etc., so that the species probability of occurrence was multiplied by the species-specific, standardised habitat amount (values 0–1) in each square. In the final step of the process, the joined overall habitat suitability index was calculated for each of our four species groups (i.e. the mean of the forest species joined habitat suitability indices, and so forth for all species groups) in each square. The degree of matching between most optimal areas for species and the reserve network was tested using Spearman rank correlation, where the habitat suitability index of species and species groups was related to the amount of protected habitat (hereafter “proportion protected”) in each square. Because of large sample sizes (452–1539) in these correlation analyses, only very low probability values (p<0.001) can be regarded as “significant”. In comparing the overall habitat suitability index of the four species groups in 1971–2000 and in scenarios in the period 2051–2080, we used 5% hotspots, that is 5% of squares with the highest values of this index, separately for each species group in each boreal zone.

## Results

**Figure 2:**
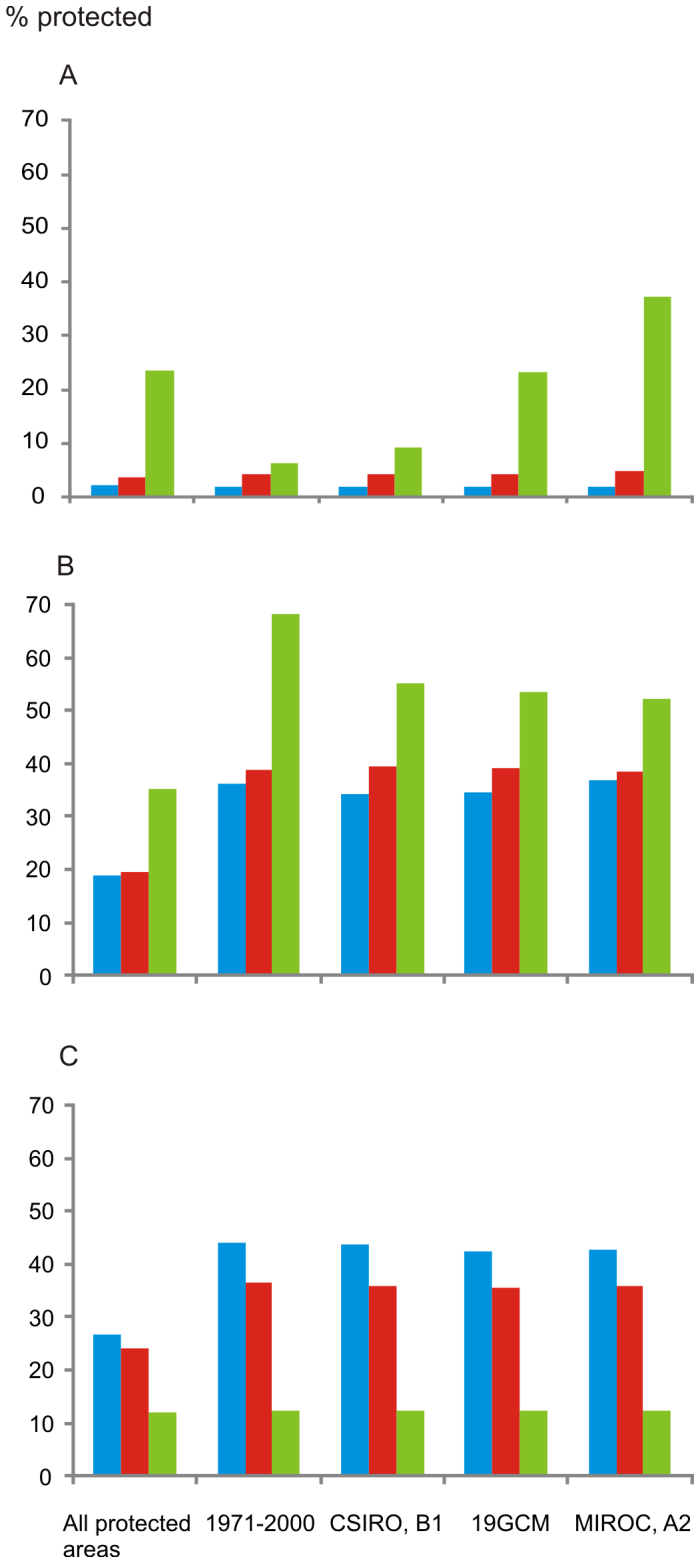
The proportion of protected habitat in 1971–2000 and in 2051–2080. Overall proportion (%) of protected habitat (All protected areas; i.e. the amount of protected habitat vs. the total amount of the habitat) and the proportion (%) protected in the 5% hotspots (1971–2000; CSIRO, B1; 19GCM; MIROC, A2) in three of the studied habitats, forests (A), mires (B) and marshlands (C), and in the three vegetation zones: blue column = the southern boreal zone, red column = the middle boreal zone, green column = the northern boreal zone. The 5% hotpots are separately determined for the three species groups based on the overall habitat suitability index of species and model projections for the observed climate in 1971–2000 and the three scenarios for 2051–2080 (B1, 19GCM ensemble mean A1B, A2).

**Figure 3:**
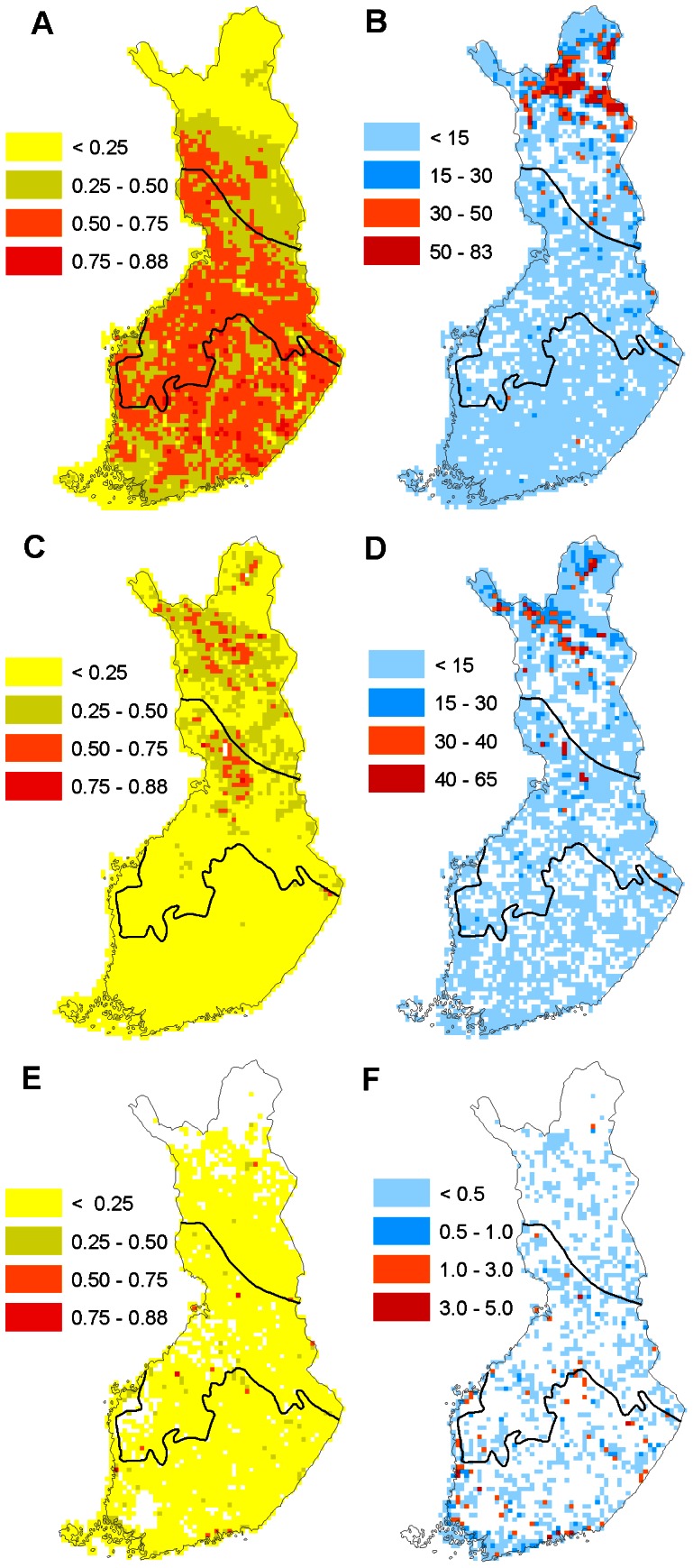
Species-specific habitat suitability index and the amount of protected habitat in the southern boreal, middle boreal and northern boreal zones in Finland. Variation in species-specific habitat suitability index of species (A, C, E) and the amount of protected forest, mire and marshland habitat (in km^2^; B, D, F; respectively) in 2051–2080 according to the 19GCM ensemble mean in a forest species (the red-breasted flycatcher *Ficedula parva*; A, B), in a mire species (the wood sandpiper *Tringa glareola*: C, D) and in a marshland species (the marsh harrier *Circus aeruginosus*: E, F).

The patterns in the overall habitat suitability index of species varied between the four species groups, the forest, mire, marshland and mountain bird species. Figure 2 shows the percentage of total protected habitat in each habitat type in the different vegetation zones, the percentage of protected habitat in the 5% hotspots of the overall habitat suitability index in 1971–2000 and under the three scenarios for 2051–2080. These results suggest that in forest species, the amount of protected forests in the 5% hotspot squares was considerably lower in the northern boreal zone in 1971–2000 compared to the average in the zone. This is due to the fact that in the northern boreal zone, forest protection is concentrated in the northernmost region, where southern forest bird species do not presently occur (Figure 3A, 3B, see also Figure 1).

**Figure 4:**
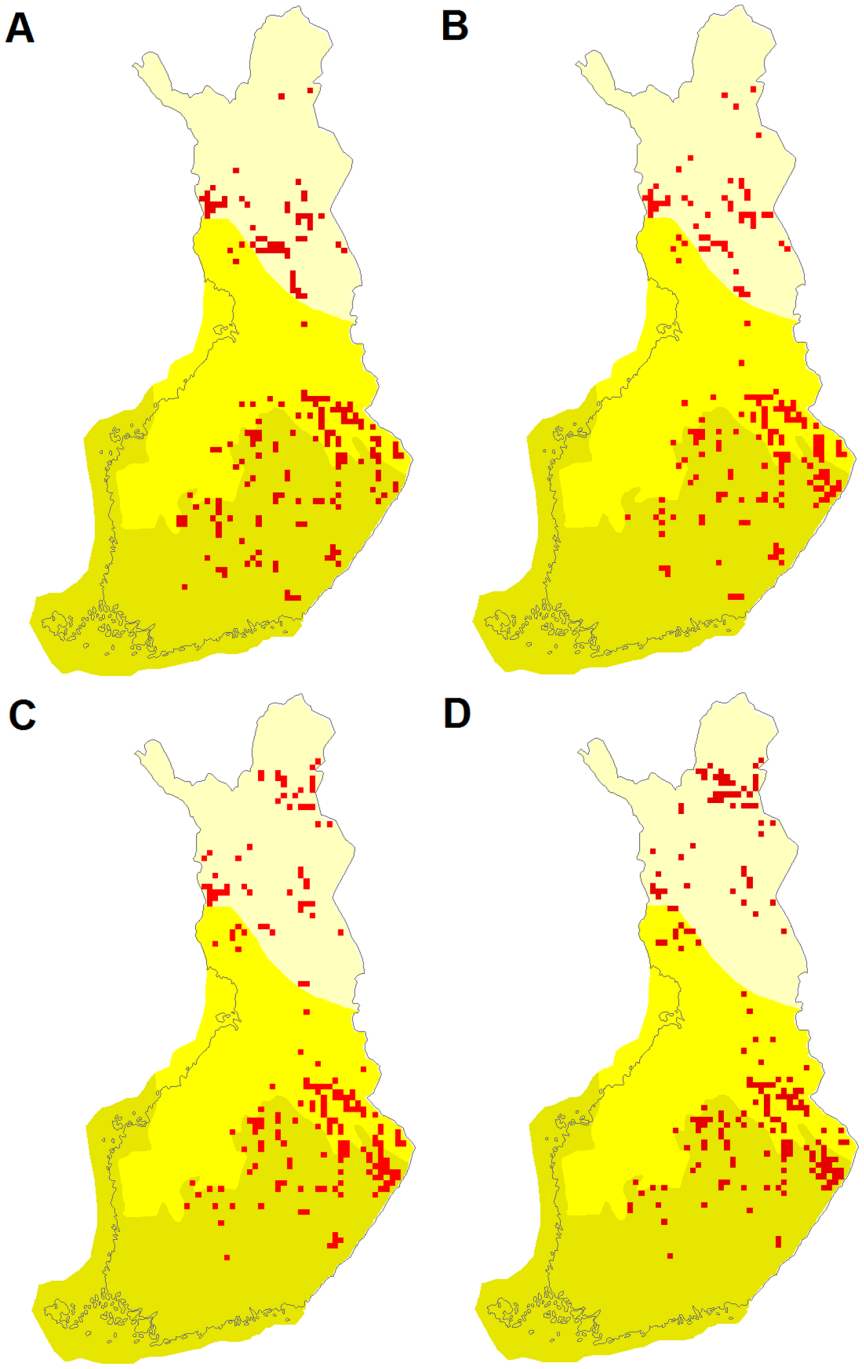
The location of the 5% hotspots of forest species. Hotspots are based on the overall habitat suitability index of species in 1971–2000 (A) and in 2051–2080 according to the three scenarios: B = CSIRO, B1; C = 19GCM ensemble mean, A1B; D = MIROC, A2. The southern boreal zone = olive, the middle boreal zone = yellow, the northern boreal zone = light yellow.

However, according to climate scenarios for 2051–2080, the amount of protected forest in the northern boreal zone increases in the 5% hotspots as species are predicted to move northwards (Figure 2 and Figure 4). According to the ensemble mean scenario, the proportion protected in 2051–2080 in the 5% hotspots is much higher than in the hotspots of 1971–2000. In the A2 scenario, the proportion protected in the 5% hotspots is six times higher than in the 5% hotspots in 1971–2000 and also higher than the present average in northern boreal forests, indicating the increasing importance of northern reserves for forest species conservation under more severe climatic changes. Geographically, the 5% hotspots are concentrated in the eastern part of the country in the southern and middle boreal zones but not in the northern boreal zone (Figure 4).

In mires, the proportion of protected habitat in the 5% hotspot squares is higher than the average in all the three zones (Figure 2), suggesting that protected mires match successfully with the most suitable sites of several mire birds (see Figure 3C, 3D). In the scenarios for 2051–2080, the proportion protected in the 5% hotspots remains the same in the southern and middle boreal zones as in 1971–2000, but in the northern boreal zone it is projected to decline. In the marshlands, the amount of protected habitat is much higher in the 5% hotspots in 1971–2000 compared to the average in the southern and middle boreal zones but not in the northern boreal zone. The proportion protected in the 5% hotspots in the marshlands is projected to remain the same in all zones in the future (Figure 2, see also Figure 3E, 3F).

In the mire bird species as a group, there is a significant (p<0.001) positive correlation between the amount of protected habitat and the overall habitat suitability index of bird species in all the scenarios and in all the boreal zones, as there is with marshland birds in the southern and middle boreal zones and Arctic mountain birds in the northern boreal zone (Table 1). In the forest species there is significant (p<0.001) positive correlation only in A2 scenario in the middle boreal zone.

**Table 1:** Correlation (Spearman rank, r_s_) between the amount of protected habitat and overall habitat suitability index of species groups in a given square.

Species group	Scenario	Southern boreal		Middle boreal		Northern boreal	
		r_s_	p	r_s_	p	r_s_	p
Forest species (51)	CSIRO, B1	0.058	0.022	0.076	0.010	−0.051	0.094
	19GCM, A1B	0.057	0.025	0.101	0.001	0.040	0.194
	MIROC3, A2	0.057	0.026	0.123	<0.001	0.093	0.002
Mire species (12)	CSIRO, B1	0.363	<0.001	0.517	<0.001	0.339	<0.001
	19GCM, A1B	0.364	<0.001	0.523	<0.001	0.332	<0.001
	MIROC3, A2	0.380	<0.001	0.523	<0.001	0.324	<0.001
Marshland species (17)	CSIRO, B1	0.484	<0.001	0.295	<0.001	0.108	0.007
	19GCM, A1B	0.480	<0.001	0.300	<0.001	0.092	0.020
	MIROC3, A2	0.472	<0.001	0.303	<0.001	0.099	0.013
Species of Arctic mountain heaths (9)	CSIRO, B1	–	–	–	–	0.825	<0.001
	19GCM, A1B	–	–	–	–	0.825	<0.001
	MIROC3, A2	–	–	–	–	0.825	<0.001
Species of Arctic mountainbirch woods (2)	CSIRO, B1	–	–	–	–	0.866	<0.001
	19GCM, A1B	–	–	–	–	0.861	<0.001
	MIROC3, A2	–	–	–	–	0.865	<0.001

The overall habitat suitability index was based on the mean of species-specific habitat suitability indices, which is the probability of species occurrence in 2051–2080 multiplied by the amount of habitat preferred by species in each square. The amount of habitat in this calculation was scaled in each square by dividing it by the largest amount of the given habitat in each zone. The probability of species occurrence was based on three different climate scenarios: B1 (CSIRO), ensemble mean of 19 GCM based on A1B (19GCM) and A2 (MIROC3). Squares in which the given habitat was absent were excluded from the correlation analyses. The number of squares included in each species group, in southern, middle and northern boreal zones, respectively: in species of forests 1539, 1164, and 1071, in species of mires 1509, 1159, and 1069, and in species of marshlands 1313, 989, and 636. The number of squares included in Arctic mountain heaths was 686 and in Arctic mountain birch woods 452. The number of species in each species group in parentheses.

The results of species-specific comparisons based on the ensemble scenario are similar to those of the overall habitat suitability index of species groups (Table S2): all correlations between the habitat suitability index of species and the amount of protected habitat are significant (p<0.001) and positive in all mire species in all zones, in all marshland species in the southern and middle boreal zones and in all species of Arctic mountain habitats in the northern boreal zone. In contrast, in forest species the correlations vary between species: in the southern boreal zone the correlations are significant (p<0.001) and positive in only three species, and in the middle boreal zone significant and positive in 21 species, and significant and negative in two species. In the northern boreal zone there are more significantly (p<0.001) negative (12) than positive correlations (9). Southern species do not reach the northernmost part of the northern boreal zone, and thus their habitat suitability indices correlate negatively with protected habitat (for example the black stork *Ciconia nigra*, the lesser spotted eagle *Aquila pomarina*, the white-backed woodpecker *Dendrocopos leucotos*, the red-breasted flycatcher *Ficedula parva* (see also Figure 3A), the collared flycatcher *F. albicollis* and the marsh tit *Parus palustris*). In contrast, the probability of the occurrence of northern species of forests is highly positively correlated with a protected habitat (for example the hawk owl *Surnia ulula*, the great grey owl *Strix nebularia*, the Siberian tit *Parus cinctus*, the Siberian jay *Perisoreus infaustus*, the two-barred crossbill *Loxia leucoptera* and the pine grosbeak *Pinicola enucleator*).

## Discussion

### Species Hotspots and Conservation Planning under Changing Climate

Many of the earlier diversity hotspots studies have focused either on examining the spatial variation in patterns of species richness [37], [39], [82], the coincidence of hotspots in different taxa [38], or modelling of the relationships between species richness and environment at broad (e.g. [83], [84]) or regional [85]–[87] scales. Such studies provide important insights and preservation of diversity concentrations, but it is increasingly argued that longer-term planning needs to take into account the impacts of climate change on the diversity hotspots [31] and species-based conservation activities [14].

One commonly used approach for assessing the impacts of climate change on the efficiency of reserve networks is the use of bioclimatic envelope models to predict spatial changes in species distributions, and then relate these projections to the present-day structure of the network. In such studies the probabilities of species occurrences are often converted into plain presence/absence forecasts that can be employed to assess the turnover of species in protected areas [13], [29], [30], [33], or combined for different areas to determine future richness hotspots which are not covered by the present reserve network [11], [41] or fed into reserve selection algorithms to examine whether new protected areas are required to maintain species representation at an adequate level [10]. Some climate change impact studies have specifically focused on projected changes in diversity hotspots, but this is usually done by employing forecasts of species presences/absences to assess species’ future range expansions and contractions [40], [42], [43], [88]. Such studies share the same potential shortcoming: the loss of information resulting from the conversion of probability values into plain presence/absence data, resulting in limited possibilities for comparing the forcefulness of the projected suitability changes between different areas.

Recent studies have made important steps towards sharpening how projections from species climate-impact models are utilized when assessing the efficiency of the reserve network. As regards the problem of assessing species’ conservation status in grid cells with different amounts of protected surface area, an important recent improvement was the development of the joint index that integrates the projected climatic suitability of the cell (i.e. probability of occurrence) with the proportion of the cell that is conserved [14], [34]. In the field of climate change impacts on diversity hotspots, Milanovich et al. [31] discussed the potential shortcomings of using only one threshold for developing species presence/absence projections and employed two different cut-off levels, whereas McClean et al. [89] used species projections with an 11-point probability of occurrence scale to identify sets of grid cells in an African plant priority area for the species in the future. One of the most important studies is that of Marini et al. [35], where the authors used a wide range of models to generate species-specific cumulative (from the ensemble of 48 separate models) future projections to detect potential gaps in the reserve system for Brazilian Cerrado biome birds. The study culminates in the identification of areas where high future probabilities of occurrence coincide with several species, and the comparison of these hotspots with the current reserve system.

In the present study, we also made an attempt to sharpen the BEM-based assessments of the efficiency of the reserve network by taking the predicted probabilities of occurrence as a continuous factor into account, but by integrating some hitherto very rarely applied measures in the process. This was done by considering the amount of protected habitat preferred by the study species in each grid cell (not just the total protected area), developing a joint habitat suitability index for each grid cell and species by multiplying the climatic suitability predictions with the cover of suitable habitat in the cell, and ultimately by combining the species-specific suitability values across all species to produce an overall suitability index for the species groups representing four major habitats. Without doubt, our study has limitations of its own, and it could be developed further by including more than one time slice into which the models are projected (e.g. [10], [41]), wider sets of climate predictor variables [90], more modelling methods [35], [40] and model selection criteria and envelope uncertainty assessments [86], [91], for example, but such dimensions are beyond the scope of this work. By and large, the key methodological point put forward in this study is that instead of using plain presence/absence predictions, we should aim to increase the accurateness of the climate change-based assessments on species range changes vs. reserve networks. The consideration of a preferred habitat for the study species in and outside reserves and the integrated habitat suitability index employed here have the potential to provide an important tool for the enhanced analysis of the efficiency of the reserve system.

### Patterns of Efficiency of Protected Areas

In a pan-European study, Araújo et al. [14] found that a large proportion (58%) of European plant and terrestrial vertebrate species may lose more suitable climate space in presently occupied protected areas than they gain in new areas by 2080. However, so far only a few regional or national studies dealing with the effects of predicted climate change on the biodiversity of protected areas have been carried out in Europe [92]. Our detailed analyses of Northern Europe shows that there is a significant between-habitat variation in the patterns of efficiency of the reserve network to preserve species in a future changing climate. There is also variation in the projected efficiency of the reserve network depending on the climate scenario, which in Northern European conditions is largely related to the velocity of climate warming.

Our results showed high correlations between the species-specific and overall habitat suitability index and the amount of protected focal habitat, which is particularly visible in the high proportion of protected habitat in the 5% hotspot squares based on the overall habitat suitability index for mire and marshland birds. It seems that the present reserve network is efficient or fairly efficient for the mire bird species in all boreal zones and under all the studied climate scenarios, for the marshland species in the southern and middle boreal zones, and for the Arctic mountain species in the northern boreal zone. This success reflects the fact that the conservation efforts of mires and marshlands have been largely targeted at areas where there are large concentrations of these ecosystems, especially in the southern and middle boreal zones.

On the other hand, the protected area network for forests appears not to be as efficient as in the other habitats in preserving bird species of conservation concern under a changing climate. Neither the species-specific habitat suitability index of individual species nor the overall suitability index for forest species group positively correlated to any great extent with the occurrence of present protected areas. Therefore, in forests the protected area network is far from adequate for ensuring the survival of forest bird species of conservation concern in a changing climate. In the southern and middle boreal zones in particular the cover of protected areas is clearly too low (2.3–3.7% of forest protected) to provide efficient protection. These findings echo similar concerns put forward in a number of climate change - biodiversity impact assessments. A key point in earlier studies has been that climate change increases the demand for the extent of the reserve network (see, e.g. [6], [93]) because species have to disperse across fragmented, human-modified landscapes due to northward range shifts [94], and protected areas facilitate species’ range expansions [95].

However, in the northern boreal zone, a much larger proportion of forests are protected, but protected areas are largely concentrated in the northernmost part of the northern boreal zone. Due to the concentration of protected areas in the northern boreal zone (see Figure 3B and Figure 4), the efficiency of protected areas to preserve forest species is increasing with more pronounced climate warming, under scenario A2. However, this increase does not apply to all the forest bird species studied. In particular, under the A2 scenario the northern species of forests are predicted to decrease the most. Therefore, the distribution patterns of species (e.g. southern or northern) in a given habitat are crucial in analyzing the efficiency of a protected area network to preserve species in the focal habitat.

The importance of acknowledging the differences in species’ geographical ranges has also been demonstrated in the realized population trends in bird species in North Europe. For example, Virkkala and Rajasärkkä [96] observed that the population density of southern species had increased and northern species decreased in Finnish protected areas from 1981–1999 to 2000–2009. Moreover, there was a northward density shift of species in protected areas, as the population density of species distributed over the whole country had increased in northern protected areas and decreased in southern protected areas [97]. In agreement with this, Brommer et al. [98] used broader-scale data from three nationwide bird atlases of Finland to demonstrate that the latitudinal centres of distributions of southern bird species show a clear and significant range shift northwards, and that there is also a tendency for a similar (but not statistically significant) shift in birds with northern ranges. A recent paper [99] further illustrated the potential dangers of deriving confounded results from climate change impact studies when too little attention is paid to the effects of the species’ present-day geographical ranges on the results. Hof et al. [99] reported that the (sub)arctic mammal species in Europe may benefit rather than face increased stress from future climate change, potentially leading to an increased species richness of mammals in northernmost Europe. The problem in this study is that it does not properly separate species with different present-day ranges, and thus mixes the few true Northern European Arctic and subarctic species (e.g. the Arctic fox *Alopex lagopus* and the Norway lemming *Lemmus lemmus*) with the majority of species which either have large European-wide ranges or which occur predominantly in hemiboreal or temperate regions in Central Europe (e,g, the red fox *Vulpes vulpes* and the European rabbit *Oryctolagus cuniculus*), and with some introduced alien species (e.g. the American mink *Neovison vison*). Given this, the discrepancies between the findings by Hof et al. [99] and the earlier studies [49], [51] are not surprising and can be explained by the delimitation of the species set used in the climate change impact modelling and the way the species present-day ranges are acknowledged.

In general, climate change effects on the bird species of boreal and Arctic habitats are largely habitat-specific, with large differences in response times and susceptibility [100]. The different patterns between mires, marshlands and forests are partly due to the fact that the assemblage of bird species of conservation concern is more variable in forests. Almost all bird species of marshlands are southern and all species of mires and Arctic mountains are northern, whereas bird species of forests include both southern and northern species. Thus, the acknowledgement of the variation in distribution patterns both within and between species groups with different habitat preferences is highly important in the assessments of efficiency of protected area networks to preserve biodiversity in the face of climate change.

The Aichi biodiversity target for 2020 agreed at the Nagoya summit for the extent of protected areas is at least 17% of land area. Interestingly, our results with bird species of conservation concern are partly parallel with this target, as habitats having an efficient buffer against climate change, mires, marshland and Arctic mountain habitats had an overall higher proportion that was protected, 28%, 25% and over 80%, respectively. This issue, however, is not as straightforward as one might think, because the northern boreal zone, with 23% of forests protected, does not properly preserve forest species according to the different scenarios, due to the unequal location of protected areas and the variation in the forest species distribution pattern.

### Adaptation to Climate Change

The protected area network is part of a climate adaptation strategy to preserve biodiversity in a changing climate [6], [10], [101], [102]. In general, climate adaptation strategies to preserve biodiversity can be divided into resistance and resilience strategies. Resistance strategies attempt to maintain the status quo of biodiversity in the face of climate change, and resilience strategies to enhance species and ecosystems to accommodate disturbances caused by changing climate [101], [103]. Restoring degraded habitats (see also [6]) can be included in resistance strategy, whereas resilience strategies might include translocating species beyond current range limits or actively creating new corridors to allow species movement.

The protected area network in Finnish mires, marshlands or Arctic mountain habitats is not situated at suboptimal sites in relation to predicted climate change, as measured by the probabilities of occurrences of bird species’ conservation concern and the hotspots of the most suitable areas for several species. In the future, protected areas seem to cover a fairly high or high proportion of occurrences of bird species, on average, in these habitats according to all scenarios, with only some slight decreases in the hotspots of predicted species occurrences. However, protected areas cannot ultimately prevent the predicted decrease in the northern species of mires, forests and Arctic mountain habitats [20], [24], [49] if the climate space for these species disappears. Both degraded mire habitats and forest habitats in terms of biodiversity have been restored in Finnish protected areas in a large programme [104], [105], which also benefits the future survival of bird species in these habitats.

In the forests of the southern and middle boreal zones, the proportion of habitat protected and the predicted habitat suitabilities of bird species in the 5% hotspots are so low that protected areas are likely to have only a limited impact on the species’ resistance to climate change. Our suitability hotspots results for forest birds suggest that the protected area network would benefit from its enlargement, particularly in the north-eastern part of the southern boreal zone and in the south-eastern and north-western part of the middle boreal zone.

In the northern boreal zone the extent of protected areas is much higher, but due to the concentrated location of the areas, the significance of protected areas to preserve forest bird species of conservation concern only seems to come up in more advanced climate warming, particularly according to scenario A2. The concentrated forest protection in the northern boreal zone seems to raise an interesting question: in a mild climate change scenario, the protected area network is clearly inefficient, while in a strong climate change scenario it preserves, on average, species occurrences much more effectively (see, however, the disappearance of northern species, above). Thus, there is no single solution to preserving biodiversity in a changing climate – several future pathways should be considered (see [102]).

In conclusion, the location of future protected areas should be planned in detail in relation to predicted climate change, although in the boreal zone, species have fairly large ranges and therefore species might not be so susceptible to the smaller-scale spatial variation of protected areas (see [60]). A good example of the effects of the location of protected areas under climate change is the northern boreal zone in Finland, where forests have not been adequately protected, although almost a quarter of the forests are protected.

## Supporting Information

Table S1 Studied species of conservation concern in different classifications. Studied species of conservation concern in different classifications. DIR = EU Birds Directive species (Annex I), SPEC = species of European conservation concern (unfavourable conservation status: SPEC1–SPEC3), IBA = species of Arctic or boreal biome, EU = threatened species in European Union (unfavourable conservation status), RES = species of special responsibility in Finland, RED = red-listed species in Finland in 2010 (near-threatened and threatened specie), PREF = species preferring boreal old-growth or mature (coniferous or deciduous) forests. In parentheses species habitat preference class: F = species of forest, MI = species of mire, MA = species of marshland, AH = species of Arctic mountain heath, AB = species of Arctic mountain birch wood. * Species not breeding in Finland, but occurs in regions to the south or south-east of Finland (see text).
(DOC)

Table S2 Correlation (Spearman rank, r_S_) between the amount of protected habitat and the species-specific habitat suitability index in the given square. Correlation (Spearman rank, r_S_) between the amount of protected habitat and the species-specific habitat suitability index in the given square. The species-specific habitat suitability index is the probability of species occurrence in 2051–2080 multiplied by the amount of habitat preferred by species in each square. The amount of habitat in this calculation was scaled in each square by dividing it by the largest amount of a given habitat in each zone. The probability of species occurrence was based on the ensemble mean of 19GCM. Squares in which the given habitat was absent were excluded from the correlation analyses. The number of squares included in each species group, in southern, middle and northern boreal zone, respectively: in species of forests 1539, 1164, and 1071, in species of mires 1509, 1159, and 1069, and in species of marshlands 1313, 989, and 636. The number of squares included in Arctic mountain heaths was 686 and in Arctic mountain birch woods 452. Correlations were not calculated if the highest value of species probability index was less than 0.01.
(DOC)
