# Supplementary material for: Climate Change, Northern Birds of Conservation Concern and Matching the Hotspots of Habitat Suitability with the Reserve Network
Source: PLoS One. 2013 May 20;8(5):e63376. doi: 10.1371/journal.pone.0063376 (PMC3659043; doi:10.1371/journal.pone.0063376)
Supplement: Table S1 — Studied species of conservation concern in different classifications. Studied species of conservation concern in different classifications. DIR = EU Birds Directive species (Annex I), SPEC = species of European conservation concern (unfavourable conservation status: SPEC1–SPEC3), IBA = species of Arctic or boreal biome, EU = threatened species in European Union (unfavourable conservation status), RES = species of special responsibility in Finland, RED = red-listed species in Finland in 2010 (near-threatened and threatened specie), PREF = species preferring boreal old-growth or mature (coniferous or deciduous) forests. In parentheses species habitat preference class: F = species of forest, MI = species of mire, MA = species of marshland, AH = species of Arctic mountain heath, AB = species of Arctic mountain birch wood. * Species not breeding in Finland, but occurs in regions to the south or south-east of Finland (see text). (DOC) [file pone.0063376.s001.doc]

Table S1. Studied species of conservation concern in different classifications. DIR = EU Birds Directive species (Annex I), SPEC = species of European conservation concern (unfavourable conservation status: SPEC1–SPEC3), IBA = species of Arctic or boreal biome, EU = threatened species in European Union (unfavourable conservation status), RES = species of special responsibility in Finland, RED = red-listed species in Finland in 2010 (near-threatened and threatened specie), PREF = species preferring boreal old-growth or mature (coniferous or deciduous) forests. In parentheses species habitat preference class: F = species of forest, MI = species of mire, MA = species of marshland, AH = species of Arctic mountain heath, AB = species of Arctic mountain birch wood. * Species not breeding in Finland, but occurs in regions to the south or south-east of Finland (see text).

____________________________________________________________________________________________________________________

Species DIR SPEC IBA EU RES RED PREF

____________________________________________________________________________________________________________________

Hazel grouse *Tetrastes bonasia* (F) + – – + – – –

Willow grouse *Lagopus lagopus* (MI) – – – + – + –

Rock ptarmigan *L. muta* (AH*)* – – – + – – –

Black grouse *Tetrao tetrix* (F) + + – + + + –

Capercaillie *T. urogallus* (F) + – – + + + +

Great bittern *Botaurus stellaris* (MA) + + – + – – –

*Little bittern *Ixobrychus minutes* (MA) + + – + – – –

*Black stork *Ciconia nigra* (F) + + – + – – –

European honey buzzard *Pernis apivorus* (F) + – – – – + –

Black kite *Milvus migrans* (F) + + – – – + +

*Short-toed snake eagle *Circaetus gallicus* (F) + + – – – – –

Western marsh harrier *Circus aeruginosus* (MA) + – – – – – –

Hen harrier *C. cyaneus* (MI) + + – + – + –

Montagu's harrier *C. pygargus* (MA) + – – – – + –

Rough-legged buzzard *Buteo lagopus* (AH) – – + – – – –

*Lesser spotted eagle *Aquila pomarina* (F) + + – + – – –

Golden eagle *A. chrysaetos* (F) + + – + – + +

*Booted eagle *Hieraetus pennatus* (F) + + – + – – –

Osprey *Pandion haliaetus* (F) + + – – – + –

Merlin *Falco columbarius* (F) + – – + – – –

Spotted crake *Porzana porzana* (MA) – – – – – + –

Little crake *P. parva* (MA) – – – – – + –

Common crane *Grus grus* (MI) + – – + – – –

Common ringed plover *Charadrius hiaticula* (AH) – – – – – + –

Dotterel *C. morinellus* (AH) + – – + – + –

European golden plover *Pluvialis apricaria* (MI) + – – + – – –

Temminck's stint *Calidris temminckii* (AH) – – + + – + –

Dunlin *C. alpina* (MI) + + – + – + –

Broad-billed sandpiper *Limicola falcinellus* (MI) – + – + + – –

Ruff *Philomachus pugnax* (MI) + + – + – + –

Jack snipe *Lymnocryptes minimus* (MI) – + + + + – –

Common snipe *Gallinago gallinago* (MA) – + – + – – –

Great snipe *G. media* (MA) + + – + – + –

Eurasian woodcock *Scolopax rusticola* (F) – + – + – – –

Black-tailed godwit *Limosa limosa* (MA) – + – + – + –

Bar-tailed godwit *L. lapponica* (MI) + – + + – – –

Whimbrel *Numenius phaeopus* (MI) – – – + + – –

Spotted redshank *Tringa erythropus* (MI) – + + + + – –

Common redshank *T. totanus* (MA) – + – + – + –

Common greenshank *T. nebularia* (MI) – – + – + – –

Wood sandpiper *T. glareola* (MI) + + – + + – –

Red-necked phalarobe *Phalaropus lobatus* (MI) + – + – – + –

Long-tailed skua *Stercorarius longicaudus* (AH) – – + – – – –

Eurasian eagle owl *Bubo bubo* (F) + + – – + + –

Snowy owl *B. scandiacus* (AH) + + + + – + –

Northern hawk owl *Surnia ulula* (F) + – – – – – –

Eurasian pygmy owl *Glaucidium passerinum* (F) + – – – + – +

Ural owl *Strix uralensis* (F) + – + – – – –

Great grey owl *S. nebulosa* (F) + – + – – – –

Short-eared owl *Asio flammeus* (MI) + + – + – – –

Boreal owl *Aegolius funereus* (F) + – – – + + –

Eurasian nightjar *Caprimulgus europaeus* (F) + + – + – – –

Wryneck *Jynx torquilla* (F) – + – + – + –

Grey-headed woodpecker *Picus canus* (F) + + – + – – +

*Green woodpecker *P. viridis* (F) – + – + – – –

Black woodpecker *Dryocopus martius* (F) + – – – – – +

*Middle spotted woodpecker *Dendrocopos medius* (F) + – – – – – –

White-backed woodpecker *D. leucotos* (F) + – – – – + +

Lesser spotted woodpekcer *D. minor* (F) – – – – – – +

Three-toed woodpecker *Picoides tridactylus* (F) + + – + + – +

Woodlark *Lullula arborea* (F) + + – + – – –

Horned lark *Eremophila alpestris* (AH) – – – + – + –

Meadow pipit *Anthus pratensis* (MI) – – – + – + –

Red-throated pipit *A. cervinus* (MI) – – + + – + –

Yellow wagtail *Motacilla flava* (MI) – – – + – + –

Bohemian waxwing *Bombycilla garrulus* (F) – – + – – – –

Bluethroat *Luscinia svecica* (AB) + – – – – + –

Common redstart *Phoenicurus phoenicurus* (F) – + – + + – –

Mistle thrush *Turdus viscivorus* (F) – – – – – – +

Savi's warbler *Locustella luscinioides* (MA) – – – – – + –

*Aquatic warbler *Acrocephalus paludicola* (MA) + + – + – – –

Great reed warbler *A. arundinaceus* (MA) – – – + – + –

Barred warbler *Sylvia nisoria* (F) + – – – – + –

Greenish warbler *Phylloscopus trochiloides* (F) – – – – – – +

Arctic warbler *Ph. borealis* (F) – – + – – + –

Wood warbler *Ph. sibilatrix* (F) – + – + – + –

Red-breasted flycatcher *Ficedula parva* (F) + – – – – – +

*Collared flycatcher *F. albicollis* (F) + – – – – – –

Bearded parrotbill *Panurus biarmicus* (MA) – – – – – + –

*Marsh tit *Parus palustris* (F) – + – – – – –

Willow tit *P. montanus* (F) – – – + – – –

Siberian tit *P. cinctus* (F) – – + – – – +

Crested tit *P. cristatus* (F) – + – – – – –

Penduline tit *Remiz pendulinus* (MA) – – – – – + –

Eurasian treecreeper *Certhia familiaris* (F) – – – – – – +

Eurasian golden oriole *Oriolus oriolus* (F) – – – + – + –

Great grey shrike *Lanius excubitor* (MI) – + – + – – –

Siberian jay *Perisoreus infaustus* (F) – + + + + + +

Brambling *Fringilla montifringilla* (F) – – + – – – –

Arctic redpoll *Carduelis hornemanni* (AB) – – + – – – –

Two-barred crossbill *Loxia leucoptera* (F) – – + – – – –

Parrot crossbill *L. pytyopsittacus* (F) – – + – + – –

Common rosefinch *Carpodacus erythrinus* (F) – – – – – + –

Pine grospeak *Pinicola enucleator* (F) – – + – + – +

Lapland longspur *Calcarius lapponicus* (MI) – – + – – – –

Snow bunting *Plectrophenax nivalis* (AH) – – + + – + –

Rustic bunting *Emberiza rustica* (F) – – + + – + –

Little bunting *E. pusilla* (MI) – – – + + – –

Yellow-breasted bunting *E. aureola* (MA) – + – – – + –

Reed bunting *E. schoeniclus* (MA) – – – + – – –

___________________________________________________________________________________________________________________
