# Supplementary material for: Climate Change, Northern Birds of Conservation Concern and Matching the Hotspots of Habitat Suitability with the Reserve Network
Source: PLoS One. 2013 May 20;8(5):e63376. doi: 10.1371/journal.pone.0063376 (PMC3659043; doi:10.1371/journal.pone.0063376)
Supplement: Table S2 — Correlation (Spearman rank, rS) between the amount of protected habitat and the species-specific habitat suitability index in the given square. Correlation (Spearman rank, rS) between the amount of protected habitat and the species-specific habitat suitability index in the given square. The species-specific habitat suitability index is the probability of species occurrence in 2051–2080 multiplied by the amount of habitat preferred by species in each square. The amount of habitat in this calculation was scaled in each square by dividing it by the largest amount of a given habitat in each zone. The probability of species occurrence was based on the ensemble mean of 19GCM. Squares in which the given habitat was absent were excluded from the correlation analyses. The number of squares included in each species group, in southern, middle and northern boreal zone, respectively: in species of forests 1539, 1164, and 1071, in species of mires 1509, 1159, and 1069, and in species of marshlands 1313, 989, and 636. The number of squares included in Arctic mountain heaths was 686 and in Arctic mountain birch woods 452. Correlations were not calculated if the highest value of species probability index was less than 0.01. (DOC) [file pone.0063376.s002.doc]

Table S2. Correlation (Spearman rank, r_S_) between the amount of protected habitat and the species-specific habitat suitability index in the given square. The species-specific habitat suitability index is the probability of species occurrence in 2051–2080 multiplied by the amount of habitat preferred by species in each square. The amount of habitat in this calculation was scaled in each square by dividing it by the largest amount of a given habitat in each zone. The probability of species occurrence was based on the ensemble mean of 19GCM. Squares in which the given habitat was absent were excluded from the correlation analyses. The number of squares included in each species group, in southern, middle and northern boreal zone, respectively: in species of forests 1539, 1164, and 1071, in species of mires 1509, 1159, and 1069, and in species of marshlands 1313, 989, and 636. The number of squares included in Arctic mountain heaths was 686 and in Arctic mountain birch woods 452. Correlations were not calculated if the highest value of species probability index was less than 0.01.

_________________________________________________________________________________

Species group Southern Middle Northern

boreal boreal boreal

_________ _________ __________

r_S_ p r_S_ p r_S_ p

_________________________________________________________________________________

Species of forests (n=51)

Hazel grouse *Tetrastes bonasia* 0.037 0.148 0.116 <0.001 0.049 0.112

Black grouse *Tetrao tetrix* 0.040 0.119 0.107 <0.001 0.052 0.087

Capercaillie *T. urogallus* 0.012 0.629 0.125 <0.001 0.068 0.027

Black stork *Ciconia nigra* 0.042 0.101 –0.080 0.007 –0.187 <0.001

European honey buzzard *Pernis apivorus* 0.083 0.001 0.088 0.003 0.006 0.848

Black kite *Milvus migrans* 0.103 <0.001 0.009 0.749 –0.181 <0.001

Short-toed snake eagle *Circaetus gallicus* 0.057 0.025 0.062 0.034 –0.009 0.767

Lesser spotted eagle *Aquila pomarina* 0.029 0.263 –0.023 0.437 –0.166 <0.001

Golden eagle *A. chrysaetos* 0.038 0.138 0.166 <0.001 0.365 <0.001

Booted eagle *Hieraetus pennatus* 0.118 <0.001 0.019 0.512 –0.022 0.472

Osprey *Pandion haliaetus* 0.047 0.064 0.089 0.002 0.049 0.106

Merlin *Falco columbarius* 0.027 0.295 0.170 <0.001 0.168 <0.001

Eurasian woodcock *Scolopax rusticola* 0.083 0.001 0.083 0.005 0.047 0.125

Eurasian eagle owl *Bubo bubo* 0.040 0.114 0.110 <0.001 0.061 0.046

Northern hawk owl *Surnia ulula* 0.073 0.004 0.205 <0.001 0.189 <0.001

Eurasian pygmy owl *Glaucidium passerinum* 0.013 0.597 0.145 <0.001 0.080 0.009

Ural owl *Strix uralensis* 0.013 0.609 0.149 <0.001 0.046 0.132

Great grey owl *S. nebulosa* 0.037 0.147 0.021 0.482 0.234 <0.001

Boreal owl *Aegolius funereus* 0.047 0.066 0.134 <0.001 0.068 0.026

Eurasian nightjar *Caprimulgus europaeus* 0.081 0.002 0.058 0.046 –0.222 <0.001

Wryneck *Jynx torquilla* 0.068 0.008 0.089 0.003 0.054 0.078

Grey-headed woodpecker *Picus canus* 0.034 0.178 0.128 <0.001 –0.041 0.176

Green woodpecker *P. viridis* 0.083 0.001 –0.010 0.738 –0.065 0.033

Black woodpecker *Dryocopus martius* 0.079 0.002 0.084 0.004 0.050 0.103

Middle spotted woodpecker *Dendrocopos medius* 0.151 <0.001 –0.143 <0.001 –0.005 0.870

White-backed woodpecker *D. leucotos* 0.027 0.291 0.140 <0.001 –0.167 <0.001

Lesser spotted woodpekcer *D. minor* 0.080 0.002 0.085 0.004 0.042 0.168

Three-toed woodpecker *Picoides tridactylus* 0.004 0.883 0.160 <0.001 0.079 0.009

Woodlark *Lullula arborea* 0.086 0.001 0.009 0.757 –0.218 <0.001

Bohemian waxwing *Bombycilla garrulus* – – 0.185 <0.001 0.272 <0.001

Common redstart *Phoenicurus phoenicurus* 0.081 0.001 0.084 0.004 0.062 0.043

Mistle thrush *Turdus viscivorus* 0.087 0.001 0.074 0.012 0.057 0.063

Barred warbler *Sylvia nisoria* 0.048 0.059 –0.124 <0.001 –0.246 <0.001

Greenish warbler *Phylloscopus trochiloides* 0.044 0.086 0.040 0.168 –0.274 <0.001

Arctic warbler *Ph. borealis* 0.037 0.144 0.191 <0.001 –0.008 0.794

Wood warbler *Ph. sibilatrix* 0.084 0.001 0.082 0.005 0.025 0.417

Red-breasted flycatcher *Ficedula parva* 0.077 0.003 0.019 0.511 –0.253 <0.001

Collared flycatcher *F. albicollis* 0.052 0.042 –0.015 0.606 –0.130 <0.001

Marsh tit *Parus palustris* 0.084 0.001 0.029 0.317 –0.113 <0.001

Willow tit *P. montanus* 0.079 0.002 0.087 0.003 0.062 0.044

Siberian tit *P. cinctus* – – – – 0.454 <0.001

Crested tit *P. cristatus* 0.069 0.007 10.090 0.002 10.047 0.123

Eurasian treecreeper *Certhia familiaris* 0.082 0.001 0.080 0.006 0.043 0.156

Eurasian golden oriole *Oriolus oriolus* 0.080 0.002 –0.029 0.328 –0.255 <0.001

Siberian jay *Perisoreus infaustus* 0.067 0.009 0.210 <0.001 0.232 <0.001

Brambling *Fringilla montifringilla* 0.060 0.018 0.191 <0.001 0.099 0.001

Two-barred crossbill *Loxia leucoptera* 0.040 0.117 0.179 <0.001 0.323 <0.001

Parrot crossbill *L. pytyopsittacus* 0.047 0.068 0.138 <0.001 0.058 0.060

Common rosefinch *Carpodacus erythrinus* 0.054 0.035 0.071 0.016 0.019 0.526

Pine grospeak *Pinicola enucleator* – – 0.155 <0.001 0.500 <0.001

Rustic bunting *Emberiza rustica* 0.051 0.044 0.185 <0.001 0.069 0.025

Species of mires (n =21)

Willow grouse *Lagopus lagopus* 0.393 <0.001 0.473 <0.001 0.430 <0.001

Hen harrier *Circus cyaneus* 0.366 <0.001 0.500 <0.001 0.349 <0.001

Common crane *Grus grus* 0.337 <0.001 0.534 <0.001 0.341 <0.001

European golden plover *Pluvialis apricaria* 0.326 <0.001 0.531 <0.001 0.395 <0.001

Dunlin *Calidris alpina* – – – – 0.510 <0.001

Broad-billed sandpiper *Limicola falcinellus* – – – – 0.589 <0.001

Ruff *Philomachus pugnax* 0.392 <0.001 0.519 <0.001 0.397 <0.001

Jack snipe *Lymnocryptes minimus* 0.334 <0.001 0.437 <0.001 0.475 <0.001

Bar-tailed godwit *Limosa lapponica* – – – – 0.484 <0.001

Whimbrel *Numenius phaeopus* 0.311 <0.001 0.471 <0.001 0.480 <0.001

Spotted redshank *Tringa erythropus* – – – – 0.499 <0.001

Common greenshank *T. nebularia* 0.305 <0.001 0.437 <0.001 0.386 <0.001

Wood sandpiper *T. glareola* 0.280 <0.001 0.511 <0.001 0.346 <0.001

Red-necked phalarobe *Phalaropus lobatus* – – 0.483 <0.001 0.558 <0.001

Short-eared owl *Asio flammeus* 0.366 <0.001 0.510 <0.001 0.366 <0.001

Meadow pipit *Anthus pratensis* 0.393 <0.001 0.545 <0.001 0.343 <0.001

Red-throated pipit *A. cervinus* – – – – 0.548 <0.001

Yellow wagtail *Motacilla flava* 0.391 <0.001 0.543 <0.001 0.341 <0.001

Great grey shrike *Lanius excubitor* 0.359 <0.001 0.512 <0.001 0.370 <0.001

Lapland longspur *Calcarius lapponicus* – – – – 0.599 <0.001

Little bunting *Emberiza pusilla* – – – – 0.484 <0.001

Species of marshlands (n = 17)

Great bittern *Botaurus stellaris* 0.472 <0.001 0.298 <0.001 0.046 0.242

Little bittern *Ixobrychus minutes* 0.450 <0.001 0.268 <0.001 0.105 0.008

Western marsh harrier *Circus aeruginosus* 0.481 <0.001 0.304 <0.001 0.084 0.035

Montagu's harrier *C. pygargus* 0.473 <0.001 0.287 <0.001 0.068 0.085

Spotted crake *Porzana porzana* 0.483 <0.001 0.305 <0.001 0.058 0.147

Little crake *P. parva* 0.444 <0.001 0.269 <0.001 0.081 0.040

Common snipe *Gallinago gallinago* 0.486 <0.001 0.307 <0.001 0.111 0.005

Great snipe *G. media* 0.244 <0.001 0.268 <0.001 0.102 0.010

Black-tailed godwit *Limosa limosa* 0.478 <0.001 0.287 <0.001 0.074 0.061

Common redshank *Tringa totanus* 0.484 <0.001 0.307 <0.001 0.106 0.007

Savi's warbler *Locustella luscinioides* 0.491 <0.001 0.272 <0.001 0.083 0.035

Aquatic warbler *Acrocephalus paludicola* 0.383 <0.001 0.207 <0.001 0.078 0.048

Great reed warbler *A. arundinaceus* 0.466 <0.001 0.295 <0.001 0.061 0.123

Bearded parrotbill *Panurus biarmicus* 0.487 <0.001 0.274 <0.001 0.067 0.092

Penduline tit *Remiz pendulinus* 0.477 <0.001 0.267 <0.001 0.070 0.078

Yellow-breasted bunting *Emberiza aureola* 0.383 <0.001 0.278 <0.001 0.037 0.353

Reed bunting *E. schoeniclus* 0.486 <0.001 0.308 <0.001 0.111 0.005

Species of Arctic mountain habitats (n = 11)

Rock ptarmigan *Lagopus muta* – – – – 0.819 <0.001

Rough-legged buzzard *Buteo lagopus* – – – – 0.828 <0.001

Common ringed plover *Charadrius hiaticula* – – – – 0.822 <0.001

Dotterel *C. morinellus* – – – – 0.824 <0.001

Temminck's stint *Calidris temminckii* – – – – 0.814 <0.001

Long-tailed skua *Stercorarius longicaudus* – – – – 0.815 <0.001

Snowy owl *Bubo scandiacus* – – – – 0.814 <0.001

Horned lark *Eremophila alpestris* – – – – 0.820 <0.001

Bluethroat *Luscinia svecica* – – – – 0.863 <0.001

Arctic redpoll *Carduelis hornemanni* – – – – 0.797 <0.001

Snow bunting *Plectrophenax nivalis* – – – – 0.817 <0.001

________________________________________________________________________________
